# Computational Classification Approach to Profile Neuron Subtypes from Brain Activity Mapping Data

**DOI:** 10.1038/srep12474

**Published:** 2015-07-27

**Authors:** Meng Li, Fang Zhao, Jason Lee, Dong Wang, Hui Kuang, Joe Z. Tsien

**Affiliations:** 1 Brain and Behavior Discovery Institute and Department of Neurology, Medical College of Georgia, Georgia Regents University, Augusta, GA, 30912, USA; 2 The Brain Decoding Center, Banna Biomedical Research Institute, Xi-Shuang-Ban-Na Prefecture, Yunnan Province 666100, China

## Abstract

The analysis of cell type-specific activity patterns during behaviors is important for better understanding of how neural circuits generate cognition, but has not been well explored from *in vivo* neurophysiological datasets. Here, we describe a computational approach to uncover distinct cell subpopulations from *in vivo* neural spike datasets. This method, termed “inter-spike-interval classification-analysis” (ISICA), is comprised of four major steps: spike pattern feature-extraction, pre-clustering analysis, clustering classification, and unbiased classification-dimensionality selection. By using two key features of spike dynamic - namely, gamma distribution shape factors and a coefficient of variation of inter-spike interval - we show that this ISICA method provides invariant classification for dopaminergic neurons or CA1 pyramidal cell subtypes regardless of the brain states from which spike data were collected. Moreover, we show that these ISICA-classified neuron subtypes underlie distinct physiological functions. We demonstrate that the uncovered dopaminergic neuron subtypes encoded distinct aspects of fearful experiences such as valence or value, whereas distinct hippocampal CA1 pyramidal cells responded differentially to ketamine-induced anesthesia. This ISICA method should be useful to better data mining of large-scale *in vivo* neural datasets, leading to novel insights into circuit dynamics associated with cognitions.

One of the goals of the BRAIN Initiative is to understand how the brain generates cognition in real time and how various circuit cellular components (i.e. distinct neuron types) contribute to such cognitive computations. At the moment, the neuroscience field is undergoing a major technological transformation that allows simultaneous recordings of increasingly large numbers of neurons from rodents to monkeys and humans^1^,^2^,^3^,^4^. As such, unprecedented large amounts of neural spike datasets already exist or will be collected. There is a strong need for in-depth data mining in order to gain novel insights into various cognitive tasks and behavioral states^1^,^3^,^5^,^6^,^7^. Much of the information within large-scale spike datasets, such as the identity and types of distinct neuron subtypes, has yet to be fully explored.

Herein, we set out to establish a general computational approach capable of uncovering and profiling cell subtypes from any neural spike datasets. We set the goal for ourselves that such computation-based cell-profiling methods should produce robust cell classifications that are invariant over the different brain states, which are known to alter cell-firing rates or spike patterns. Our computational approach described here - (ISICA) - emphasized on the identification of a key set of spike-activity pattern dynamics that can reflect the distinct properties for each classified cell type. Moreover, the computation-based profiling methods should be applicable to various neuron populations within a region and across multiple brain areas. Accordingly, the present study selected two types of cells as a test bed, namely the CA1 pyramidal cells of the hippocampus and dopaminergic neurons (DA) from the ventral tegmental area (VTA) in the mouse brain. These two types of cells were chosen for computational profiling not only because of their importance in cognition, but also the widely held assumption of their apparent morphological homogeneity. In addition, the extensive knowledge on these cells accumulated in the literature^8^,^9^,^10^,^11^,^12^,^13^,^14^ can enable us to compare and evaluate the effectiveness of our method. More importantly, we applied optogenetic methods to validate the ISICA-identified DA neuron subtypes. Finally, we conducted two sets of functional analysis experiments for further characterizations of ISICA-identified cell subtypes.

## Results

### Identification of key features from spike activity patterns for unit classification

Neurons in the brain can fire in a wide frequency range and generate complex, evolving activity patterns. Such dynamic firing changes reflect the summation of the unique channel compositions and membrane excitability, ongoing synaptic inputs, and varying degrees of neural/hormonal modulation resulting from both the external and internal events^1^,^15^,^16^. Therefore, spike patterns contain a lot of information. At present, it is not clear whether and what features among *in vivo* dynamic spike patterns can be used for cell classification. We set our goal to identify a set of key features from spike patterns that would correspond to the intrinsic cell identities.

**Figure 1:**
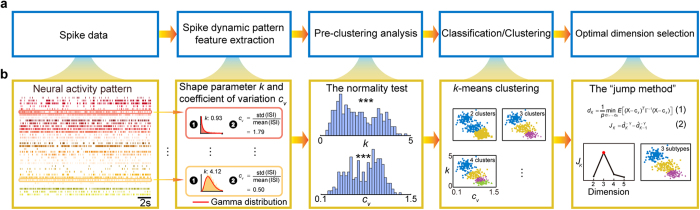
The proposed ISICA-based method. (**a**) A flow charter diagram for the ISICA-based profiling steps. (**b**) Illustration of identifying neuron subtypes using the proposed ISICA-based method.

Among multitudinous means of characterizing spike activity patterns, the inter-spike interval (ISI) is known to contain key information on several fundamental properties^17^,^18^,^19^,^20^,^21^: 1) the intrinsic properties of a neuron, especially the properties of its membrane due to the cell ontogeny; 2) neural network interactions based on how the circuits are wired during development or by experiences; and 3) the nature of external and internal inputs to the neurons, which can vary from moment to moment. From our large-scale neural recording in behaving mice, we often observed that neurons from the different brain regions - such as the prefrontal cortex (PFC), hippocampus, striatum, and VTA - exhibit diverse ISI patterns (Figure S1). This has prompted us to investigate the variations in ISI patterns as a starting point for profiling dynamic properties of the recorded neurons. We devised an Inter-Spike Interval Classification Analysis (ISICA, for short) approach to study neuron subtype profiles. This ISICA consists of four computational analysis steps (Fig. 1a): 1) feature extraction from spike dynamic patterns; 2) pre-clustering analysis on these extracted features; 3) performing classification and clustering analyses to examine neuronal feature subtypes in different dimensions. During this step, various classification/clustering methods can be used - such as k-means, spectral clustering, support vector machine (SVM) and Boosting, etc.; 4) determining the optimal dimension from which the best classification/clustering can be selected in an unbiased manner.

In the “spike-pattern feature-extraction” step, we focused on two key features: shape parameter *k* of the Gamma distribution model and coefficient of variation *c*_*v*_, to characterize the spike properties of each neuron. We used the Gamma distribution model (red curves in the second block of Fig. 1b) to describe the overall shape of a given neuron’s inter-spike interval histogram (ISIH). Gamma distribution is defined by a shape parameter *k* > 0 and a scale parameter *θ* > 0 (see Methods). Between these two parameters, *k* provides an effective way to measure the ISIH. Gamma with *k* = 1 becomes the exponential distribution, and the ISI distribution fits a Poisson process. ISI distributions with *k* > 1 indicate that the neuron discharges more frequently in short and long intervals than the exponential distribution. When *k* < 1, the peaks of ISI distributions shift away from zero. Gamma with *k* = ∞ is the distribution of no variance and the spike train is perfectly regular. We found that the ISIHs of the major neuron types, exemplified from four different brain regions; namely, the prefrontal cortex (PFC), CA1, striatum (STR), VTA, can be described by the Gamma distribution model (Figure S1b). Thus, *k* provides a parametrical measurement of spike activity patterns.

We used another feature to describe spike activity patterns, namely, the coefficient of variation (*c*_*v*_), which is a parameter-free measurement describing the spike train irregularity. In the statistics, the *c*_*v*_ is a standardized measure of dispersion of a probability distribution. In the proposed method, *c*_*v*_ is defined as the ratio of the standard deviation of the ISI to the mean of the ISI. Exponential distributions (suggesting a Poisson process) have a *c*_*v*_ of 1. ISI with a smaller *c*_*v*_ shows a more regular spike activity pattern than the Poisson process, while a larger *c*_*v*_ means that the neuron discharges more irregular spike activity pattern than the Poisson process. *k* and *c*_*v*_ are negatively correlated, that is, ISIs with a larger *k* have a smaller *c*_*v*_ and vice versa.

Upon the feature extraction, we asked if there were multiple neuron subtypes that can be identified in the recorded spike datasets. The shape parameter *k* of the Gamma distribution model and coefficient of variation *c*_*v*_ can be statistically tested for normal distribution using the D’Agostino and Pearson omnibus normality test (Fig. 1b). After non-normal distribution was detected, a set of classification/clustering analyses was conducted to unbiasedly uncover the neuron subtypes. In the present study, we chose the *k*-means clustering method to cluster the extracted features (Fig. 1b). As a popular method for cluster analysis in data mining, *k*-means clustering aims to partition *n* observations into *k* clusters in which each observation belongs to the cluster with the nearest mean, serving as a prototype of the cluster. In this classification step, a set of clustering results with different numbers of clusters can be calculated by setting a different *k*.

The final step was to determine the optimal dimension, which gives the best classification. We carried out this unbiased selection (Fig. 1b) by using the “jump method”^22^ (see Methods). The jump method determined the number of clusters that maximized efficiency while minimizing error by information-theoretical standards. Here, the jump method was used to determine the numbers of clusters in the neuron population by assessing the “distortion,” which is a measure of within-cluster dispersion [equation (1)]. The true number of clusters was selected as the sharpest jump of the distortion curve [equation (2)].

To test our ISICA computational classification approach, we used neural spike activity datasets recorded from the hippocampal CA1 region and VTA in freely behaving mice during awake and sleep periods, as well as under general anesthesia or episodic experiences^5^,^23^,^24^. We investigated on CA1 pyramidal cells and VTA DA neurons, respectively, as examples to demonstrate the usefulness of this computational-profiling approach.

### Profiling of CA1 pyramidal cell subtypes based on the ISICA method during the awake period

**Figure 2:**
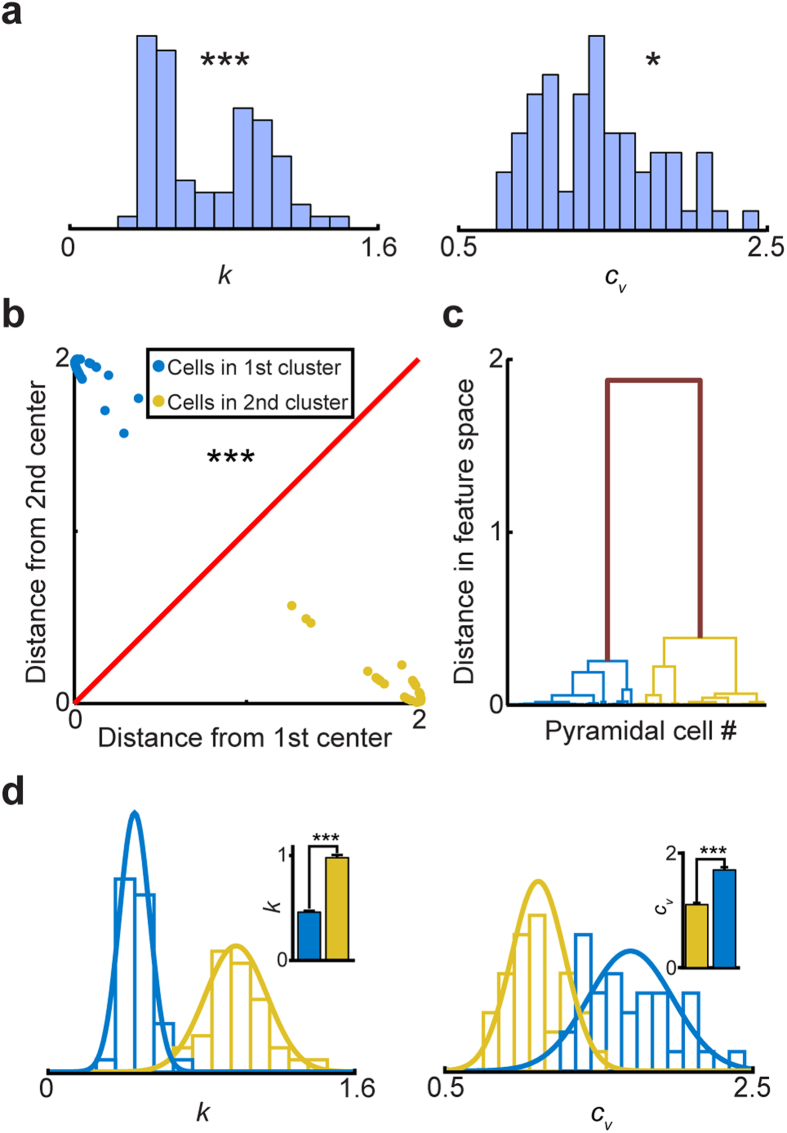
Profiling hippocampal CA1 pyramidal cells based on neural activity patterns under the awake state. (**a**) Distributions of *k* and *c*_*v*_ during the quiet awake state. *p* values from the D’Agostino and Pearson omnibus normality test indicated that there are discrete sub-populations within hippocampal CA1 pyramidal cell population. (**b**) Distances from two cluster centers revealed a significant separation of two pyramidal cell subtypes. (**c**) A hierarchical clustering analysis showed that the inter-cluster distance of two clusters was significantly higher than the intra-cluster distance. (**d**) Distributions of *k* and *c*_*v*_ for two pyramidal cell subtypes. The bar graphs showed that these two pyramidal cell subtypes had significant differences in *k* and *c*_*v*_.

We first examined spike dynamics and ISI profiles of hippocampal CA1 pyramidal cells. The neural datasets were obtained from two behavioral states - namely, the quiet awake period and the slow-wave sleep (SWS) period. The CA1 region of the hippocampus plays a crucial role in long-term memory formation^25^. While the inhibitory CA1 interneurons have been long-recognized to be very diverse^26^,^27^, CA1 pyramidal cells have been traditionally considered as a morphologically homogenous population. However, recent *in vitro*^28^ and *in vivo* experiments^29^, as well as anatomical studies^30^, suggest that CA1 pyramidal cells may not come as a homogenous population, as widely thought. Therefore, we investigated how the ISICA method would profile CA1 pyramidal cells using the spike datasets collected under the quiet awake period. A total of 70 well-separated CA1 putative pyramidal cells from three mice were used for the present analysis of extracting ISI features (see Methods). As shown in Fig. 2a, *p* values of the D’Agostino and Pearson omnibus normality tests showed that both *k* and *c*_*v*_ were not unimodally distributed, suggesting that there were multiple sub-populations of CA1 pyramidal cells. From the *k*-means clustering method and the jump method, our analyses suggested two well-separated, pyramidal cell sub-populations (Fig. 2b). The numbers of pyramidal cells in these two distinct clusters were 36 (blue dots in Fig. 2b) and 34 (yellow dots in Fig. 2b), respectively. Also, we used a bootstrap analysis to verify that the data were indeed best represented by two clusters (see Methods). Finally, hierarchical clustering analysis further showed that the inter-cluster distance of two clusters was significantly higher than the intra-cluster distance (Fig. 2c). These two neuron sub-populations showed no significant difference in their waveform (*t*-test, Figure S7a). As expected from the clustering, these two pyramidal cell sub-populations had significant differences in *k* (0.460 ± 0.013 vs. 0.983 ± 0.027, *p* < 1E-26) and *c*_*v*_ (1.705 ± 0.046 vs. 1.100 ± 0.030, *p* < 1E-15) (Fig. 2d). These results provided strong evidence for the existence of two distinct pyramidal cell sub-populations in the CA1.

### Profiling CA1 pyramidal cells under the SWS state

Since firing patterns could vary significantly in different brain states, we asked whether the ISICA-based classification remains invariant over different brain activity states. As such, we investigated the spike activity patterns of these 70 CA1 pyramidal cells using the data collected during the SWS period. The D’Agostino and Pearson omnibus normality test again suggested the non-unimodal distributions of both *k* and *c*_*v*_ features (Figure S2a). Two well-separated clusters were revealed using the *k*-means cluster analysis and the jump method. The optimality of this clustering result was verified by a bootstrap analysis (Figure S2b). A hierarchical clustering analysis further confirmed that these two clusters were well-separated (Figure S2c and S2d).

**Figure 3:**
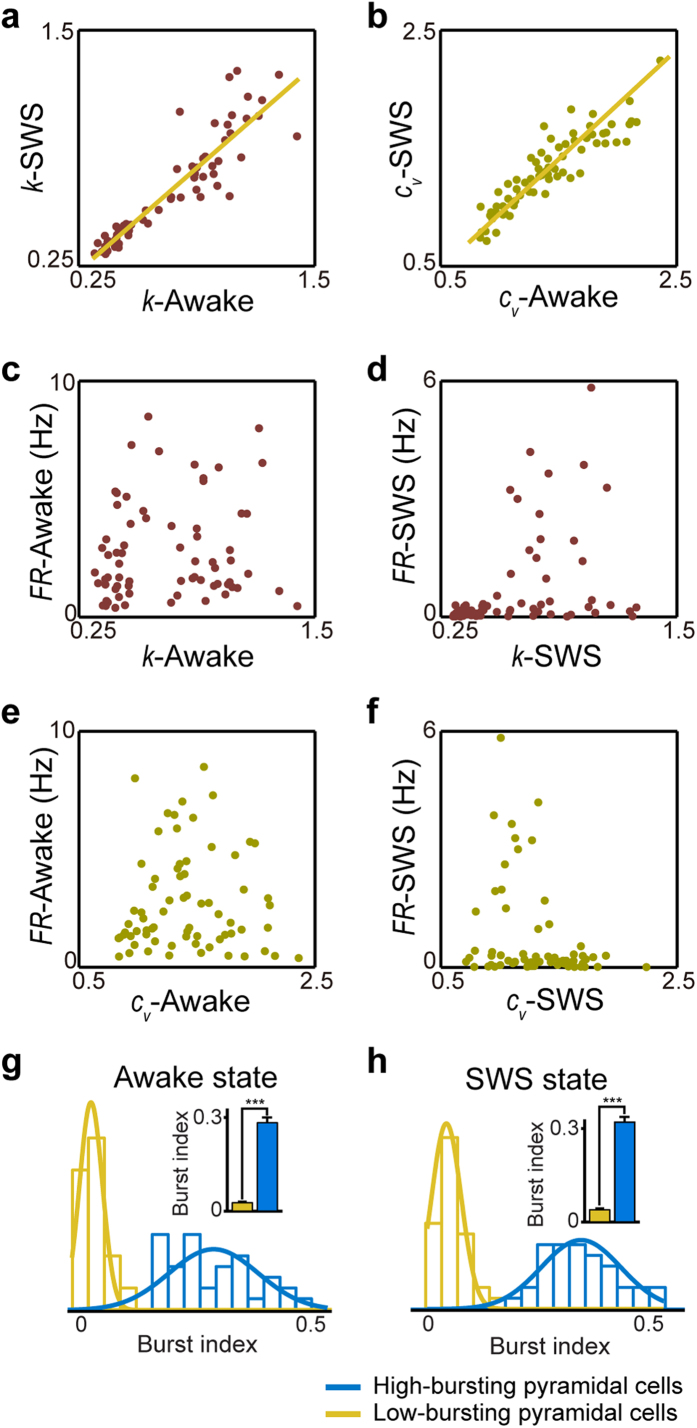
*k* and *c*_*v*_ were robust across different brain states. (**a**) *k* showed linear relation under the awake and SWS states. (**b**) *c*_*v*_ showed linear relation under awake and SWS states. (**c**,**d**) *k* was independent of neuron’s mean-firing rates under the awake or SWS states. (**e**,**f**) *c*_*v*_ was independent of neuron’s mean-firing rates under the awake or SWS two states. (**g**) Distributions of burst index of two pyramidal cell subtypes under the awake state. (**h**) Distributions of burst index of two pyramidal cell subtypes under the SWS state. The bar graphs showed that these two pyramidal cell subtypes had significant differences in burst index under two distinct brain states.

Most importantly, we observed that individual memberships of these two clusters uncovered under the SWS state were in complete agreement with those identified using the spike activity patterns recorded from the quiet awake state. In fact, *k* and *c*_*v*_ measured under these two states remained closely or proportionally matched, almost in a linear relation with linear correlation coefficients of 0.92 and 0.91 for *k* and *c*_*v*_ between the two states, respectively (Fig. 3a,b). Both features were independent of the neuron’s mean firing rates (Fig. 3c–f). The linear correlation coefficients between the mean firing rates and *k* are 0.13 under the quiet awake state (Fig. 3c) and 0.09 under the SWS state (Fig. 3d), and the linear correlation coefficients between the mean firing rates and *c*_*v*_ are 0.02 under the quiet awake state (Fig. 3e) and 0.10 under the SWS state (Fig. 3f). Taken together, these analyses suggested that the ISICA-based cell classification remained robust and invariant over different brain states.

CA1 pyramidal cells are known to burst, which is believed to be important for these excitatory principal cells to represent and process information^15^,^31^,^32^. In the literature, the burst is usually defined as the ISI shorter than 10 ms. These bursting dynamics should be intrinsically covered by shape parameters and the coefficient of variation. We measured the burst index, which is defined as the ratio of bursting ISIs to all ISIs. Since CA1 pyramidal cells tend to exhibit bursting patterns, we examined whether two pyramidal cell sub-populations, identified by the *k* and *c*_*v*_ feature extraction, would reflect or capture the difference in the burst index. We found that these two pyramidal cell subtypes indeed showed significant differences in burst indices under the quiet awake state (0.285 ± 0.016 vs. 0.027 ± 0.003, *p* < 1E-22) (Fig. 3g) and the SWS state (0.325 ± 0.014 vs. 0.040 ± 0.005, p < 1E-27) (Fig. 3h), suggesting that the ISICA method is highly sensitive to distinguish different bursting cells. For convenience in this study, we termed these two subgroups of CA1 cells, identified by this ISICA method, as the high-bursting pyramidal cells and low-bursting pyramidal cells, respectively.

### Profiling CA1 pyramidal cells under ketamine-induced anesthetized state

Since the ISICA-classified CA1 pyramidal cells possess the fundamental differences in intrinsic spike properties, we asked whether these subtypes may perform or serve different functions. To examine their functional significances, we examined how the high-bursting and low-bursting pyramidal cells reacted to ketamine-induced anesthesia. Ketamine can induce an anesthetic state referred to as “dissociative anesthesia”, that is, the patient is incapable of associating the input of afferent stimuli, and integrating information and signals to the conscious mind is reduced or blocked^33^,^34^. Dissociative anesthesia produced by ketamine has been postulated to be a result of reduced activation in the thalamocortical structures and increased activity in the limbic system and hippocampus. We compared the response characteristics of CA1 pyramidal cell subtypes between the quiet awake state and the ketamine-induced anesthetized state by measuring mean firing rates, burst indexes, the dynamic changes of the ISIHs and power-density relationships.

**Figure 4:**
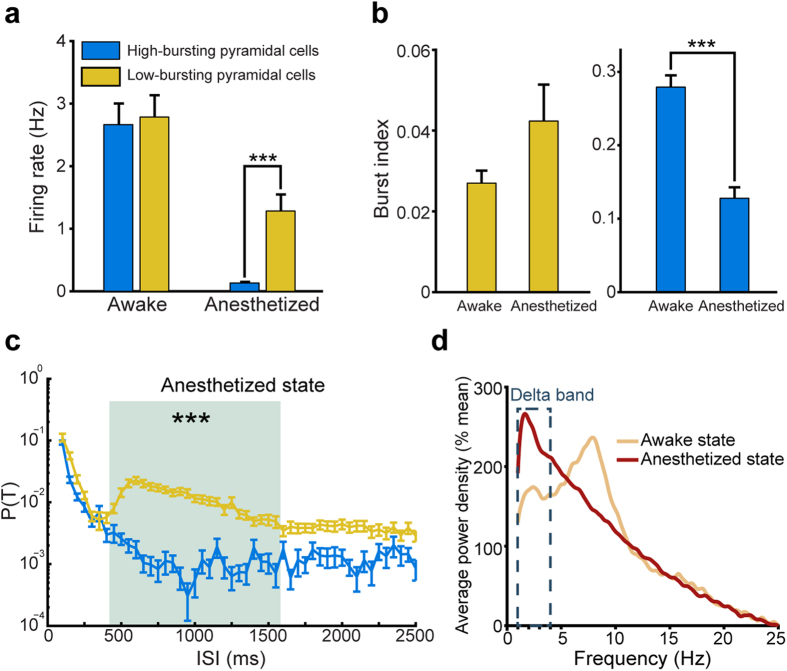
Two pyramidal cell subtypes showed significant differences upon ketamine-induced anesthesia. (**a**) Mean-firing rates of two pyramidal cell subtypes during the awake and anesthetized states. (**b**) Burst index of two pyramidal cell subtypes during the awake and anesthetized states. (**c**) ISIHs of two pyramidal cell subtypes under the anesthetized state. Asterisks and the gray region showed that the low-bursting pyramidal cells showed significantly higher firing probabilities than the high-bursting pyramidal cells under the anesthetized state. (**d**) Average local field potential power spectra under the awake and anesthetized states. The dotted box indicated Delta frequency band. Compared with the local field potential power spectra under the awake state, the anesthetized state showed significant higher power at Delta frequency band (1–4 Hz).

Under the quiet awake state there was no difference in the mean firing rates of the high-bursting and low-bursting pyramidal cell subtypes (Fig. 4a, the low-bursting pyramidal cells: 2.792 ± 0.351 Hz, the high-bursting pyramidal cells: 2.658 ± 0.362 Hz, *p* > 0.8). Interestingly, ketamine-induced anesthesia reduced firings of both cells. Yet, the high-bursting pyramidal cells were affected to a much greater degree than that of the low-bursting pyramidal cells (0.129 ± 0.024 Hz vs.1.283 ± 0.267 Hz. *p* < 1E-4). Moreover, the burst index of these two pyramidal cell subtypes changed in seemingly “opposite” directions upon ketamine-induced anesthesia (Fig. 4b). Specifically, the burst index of the low-bursting pyramidal cells appeared to show a small, but not statistically significant, increase during anesthesia (0.027 ± 0.003 vs. 0.042 ± 0.009, *p* > 0.1), whereas the high-bursting pyramidal cells exhibited a significant decrease in the burst index upon ketamine injection (0.285 ± 0.016 vs. 0.130 ± 0.016, *p* < 1E-8) (Fig. 4b). In addition, while the ISIHs of both pyramidal cell sub-populations showed no significant difference under the quiet awake state (Figure S3), the low-bursting pyramidal cells exhibited significantly higher firing probabilities at 400 ~ 1600 ms ISIs than those of the high-bursting pyramidal cells (Fig. 4c). This range of increased ISIs corresponded to the Delta frequency band (1–4 Hz)^35^, as evident from the power-density analysis (Fig. 4d). These experiments supported the notion that ISICA-classified pyramidal cell subtypes exhibited different functional regulations during ketamine-induced anesthesia.

### Profiling VTA DA neuron subtypes during the awake and sleep periods

To evaluate the general utility of the ISICA method, we then analyzed the DA neurons recorded from the VTA region of freely behaving mice during the awake period. DA neurons in the VTA are well-known to represent rewards and positive motivational information^13^,^36^. Recently, DA neurons are also found to respond to aversive stimuli^14^,^24^,^37^,^38^,^39^,^40^. These emerging results suggest that DA neurons are not a homogenous population.

A total of 53 well-separated VTA putative DA neurons were recorded and analyzed from 14 mice during the quiet awake period (see Methods). The D’Agostino and Pearson omnibus normality test suggested that there were multiple subtypes in the VTA DA neuron population (Figure S4a), and two well-separated clusters were identified by the unbiased clustering analysis (Figure S4b), with 26 and 27 DA neurons in each cluster (Figure S4c). No significant difference was observed in the waveform of these two subtypes (*t*-test, Figure S7b). As expected, *k* (1.249 ± 0.245 vs. 2.667 ± 0.513, *p* < 1E-15) and *c*_*v*_ (1.681 ± 0.330 vs. 0.705 ± 0.136 Hz, *p* < 1E-5) were significantly different between those two DA neuron subtypes (Figure S4d). Therefore, the ISICA method uncovered two distinct major subtypes of putative DA neurons.

To further examine the robustness of the ISICA method, we conducted classification analysis on the VTA DA neuron datasets recorded under the SWS state. We recorded 40 DA neurons under the SWS state from the VTA regions of 17 mice. Based on the proposed ISICA method, we also uncovered two well-separated DA neuron subtypes (18 and 27 DA neurons in each cluster) (Figure S4e–g), and those two DA neuron subtypes showed significant differences on *k* (1.307 ± 0.279 vs. 3.554 ± 0.838, *p* < 1E-10) and *c*_*v*_ (2.086 ± 0.445 vs. 0.594 ± 0.140 Hz, *p* < 1E-3) under the SWS state (Figure S4h).

**Figure 5:**
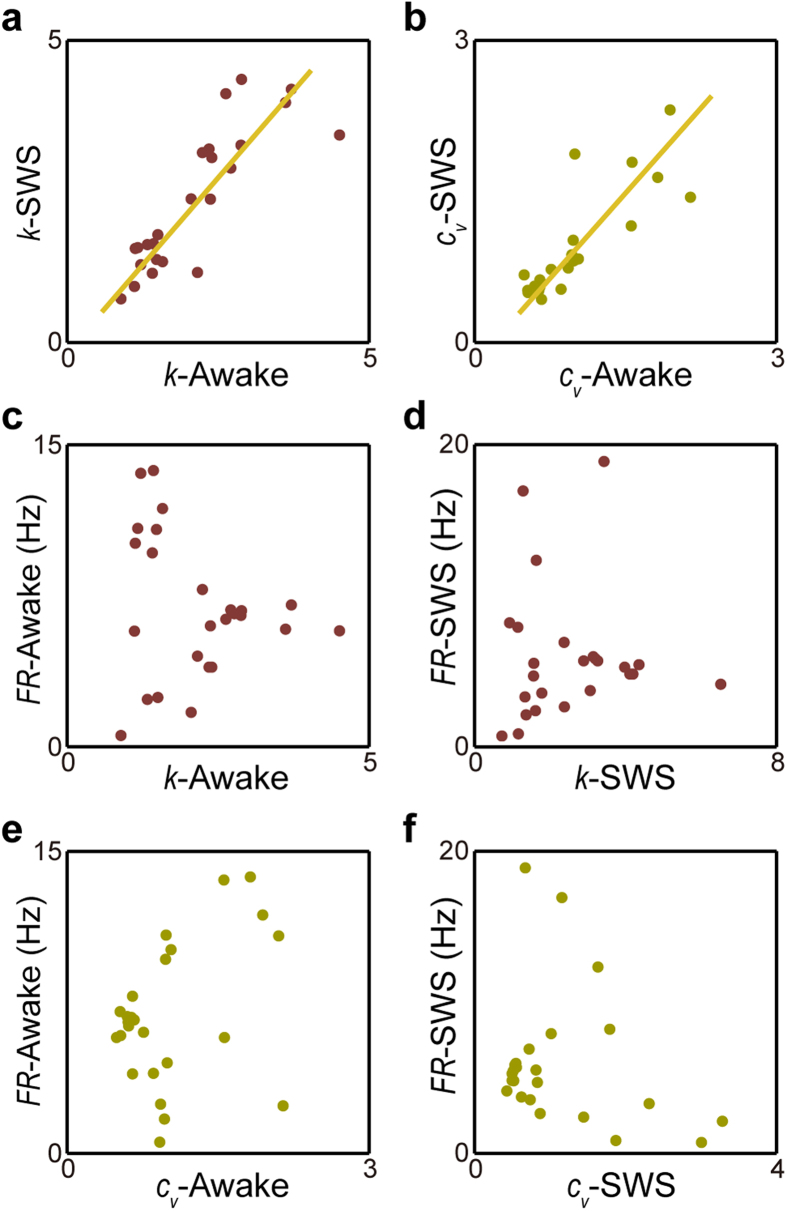
*k* and *c*_*v*_ of VTA DA neurons were tightly correlated between the awake and SWS states. *k* and *c*_*v*_ of VTA DA neurons were robust across different brain states (linear correlation coefficients of *k* and *c*_*v*_ between two states were 0.76 (**a**) and 0.72 (**b**) respectively) and were independent of neuron’s firing rates (absolute values of linear correlation coefficients were less than 0.21, (**c**–**f**). Dash lines showed the results of the linear regressions.

Out of these 40 DA neurons, 28 DA neurons were recorded in the same set of mice under the awake and the SWS states. We found that *k* and *c*_*v*_ measured under these two states remained highly consistent for these 28 units, and the linear correlation coefficients of *k* and *c*_*v*_ between the two states were 0.92 and 0.91, respectively (Fig. 5a,b). Both features were independent of the neuron’s mean firing rates (Fig. 5c–f). The linear correlation coefficients between the mean firing rates and *k* were 0.13 under the quiet awake state (Fig. 5c) and 0.09 under the SWS state (Fig. 5d). The linear correlation coefficients between the mean firing rates and *c*_*v*_ were 0.02 under the quiet awake state (Fig. 5e) and 0.10 under the SWS state (Fig. 5f). Most importantly, we found that each and every one of these 28 neurons identified under the awake state was in complete agreement of those obtained under the SWS state. Therefore, the invariant membership under the awake and sleep states, again, suggested the ISICA method had captured the key spike features for cell classification. Because *k* and *c*_*v*_ of DA neurons reflected more on the regularity of spike-activity patterns, for convenience, we termed these two subgroups of DA neurons as irregular-spiking DA neurons (smaller *k* and larger *c*_*v*_) and the regular-spiking DA neurons (larger *k* and smaller *c*_*v*_), respectively.

### Verification of VTA DA neurons using combined pharmacology and optogenetics

**Figure 6:**
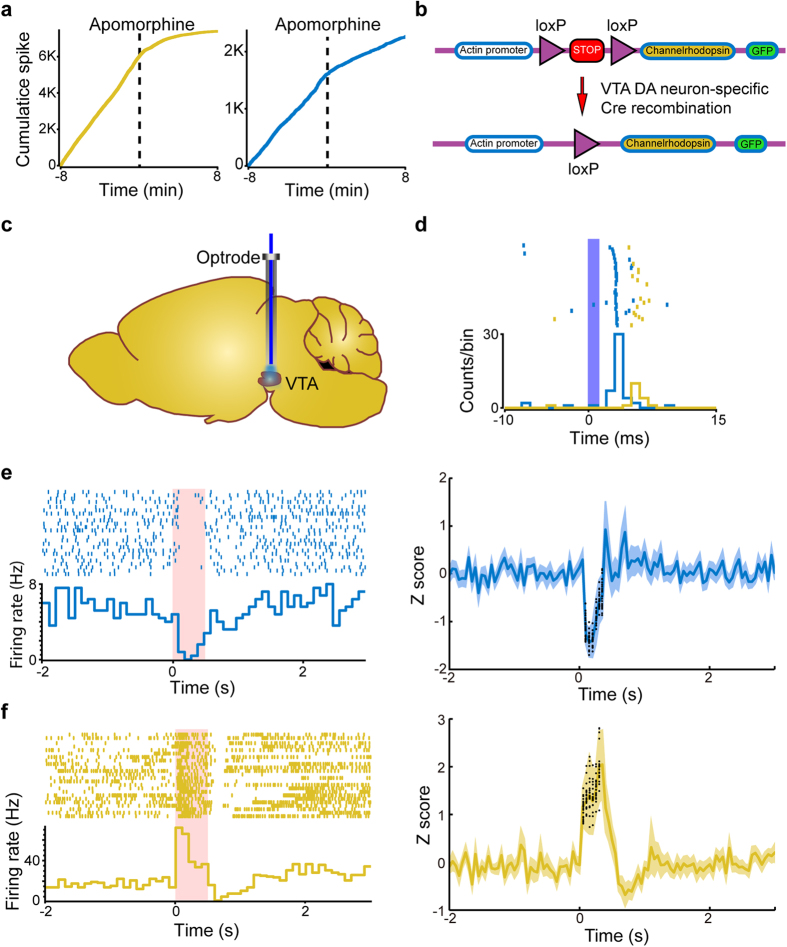
Two VTA DA neuron subtypes showed significant responsive difference under pharmacological method, optogenetics stimuli and aversive stimuli. (**a**) Cumulative spike activity of these two DA neurons in response to the apomorphine injection. (**b**) Optogenetics experiment for labeling DA neurons with ChR2. (**c**) Customized optrode was implanted and placed at the VTA. (**d**) Optically evoked neural activity of the two simultaneously recording VTA DA neurons (blue: regular-firing DA neuron, yellow: irregular-spiking DA neuron). Regular-spiking DA neurons: 12 neurons, latency: 3.74 ± 0.23 ms. Irregular-spiking DA neurons: 9 neurons, latency: 5.4 ± 0.76 ms. (**e**) Left block: the response of one representative regular-firing DA neuron upon aversive stimuli (0.5 s earthquake, as denoted by the red bars), shown as the format of perievent raster. Right block: the response of the regular-firing DA neuron population upon aversive stimuli, shown as Z score. (**f**) The response of one representative irregular-firing DA neuron and the irregular-firing DA neuron population upon fearful stimuli. In (**e**) and (**f**), the distributions of the responses of two VTA DA neuron subtypes during aversive stimuli delivery were shown as black dots.

To further verify that the members of the two clusters uncovered by the ISICA method were DA neurons, pharmacological and optogenetics methods were used in a subset of experiments. As shown in Fig. 6a, these neurons decreased their firing upon injection of D2 agonist apomorphine (1 mg/kg, *i.p.*)^24^,^41^, confirming that the neurons used in the clustering analysis were DA neurons. We then injected adeno-associated virus, coding the floxed channelrhodopsin-2 (ChR2) and green fluorescent protein (GFP), into the VTA region of a DAT-Cre mouse (Fig. 6b, see Methods). This DAT-Cre line allowed ChR2 only to be expressed in DA neurons^42^. The optrode was then implanted and placed into the VTA (Fig. 6c). We found that light-stimulation evoked time-locked activation of these two subtypes of DA neurons (Fig. 6d). Interestingly, regular- and irregular-spiking DA neurons seemed to exhibit different time latencies of optically evoked responses, with the regular-firing DA neurons at 3.74 ± 0.23 ms (*n* = 12) and the irregular-spiking DA neurons at 5.40 ± 0.76 ms (*n* = 9). The differences in latencies provided strong evidence that the two major DA neuron clusters, identified by the ISICA method, represent intrinsically distinct DA neuron subtypes.

### Distinct responses to fearful events by these two distinct DA neuron subtypes

Finally, we asked whether these two DA cell subgroups - namely, irregular-spiking DA neurons and regular-spiking neurons - exhibit different functional encoding properties. Accordingly, we investigated their responses to fearful or aversive events by using the earthquake and elevator-drop paradigms as described previously^24^. Interestingly, the regular-firing DA neurons suppressed their firing by aversive stimulus presentation (Fig. 6e). In contrast, the irregular-firing DA neurons increased their firing upon fearful stimuli deliveries (Fig. 6f). This experiment demonstrated that the DA subtypes, identified by the ISICA method, exhibited distinct functional responses to fearful information.

## Discussion

Traditionally, the methods in identifying cell subtypes relied on immunohistological and molecular/genetic profiling, and/or juxtacellular recording and labeling (which are usually conducted on brain slices or in anesthetized animals). Unfortunately, these methods are difficult to implement in freely behaving animals, especially for large-scale neural recording experiments. More recently, the genetically encoded opsin-based approach in combination with the previously developed Cre/loxP-mediated genetic methods^43^ have been applied to aid cell classification^44^,^45^,^46^. By and large, optogenetic classification studies relied on the popular promoters, such as CaMKII promoters, PV and SOM promoters. In most cases, it limits the optogentic labeling to one type of neuron per mouse Cre line and also to a few neurons located closer to the optic fiber tip where light can reach. As such, optogenetic approaches still remain to be improved, especially if researchers want to match the traditional cell subtype classifications described in the literature.

In the present study, we have developed the ISICA method that can be readily applied to any neural spike dataset which already exists or will be generated. Our analyses provided several important insights for this computational approach. First, we have successfully identified two key features - namely, the Gamma distribution shape factors *k* and coefficient of variation *c*_*v*_ - that can be used to achieve consistent classification across different brain states (i.e. the awake state vs. the SWS state). In the CA1, the ISICA method uncovered two distinct pyramidal cell subtypes. Interestingly, several laboratories reported the existence of two distinct pyramidal cell groups receiving different projections from different cortical or subcortical regions^28^,^47^. One *in vivo* study using local field potential information and vertical probes reported that CA1 pyramidal cells in the superficial layer and deep layer of the hippocampal CA1 region have distinct responses to inputs, spiking properties and output influences^29^. Intriguingly, another recent study also described that pyramidal cells throughout the CA1 and subiculum regions are topographically organized along the proximal-to-distal axis, with cells displaying the regular-spiking pattern (i.e. late-bursting cells) predominating in CA1 and the proximal subiculum and cells showing the bursting pattern (i.e. early-bursting cells) predominating in the distal subiculum^48^. At the moment, it is not clear how such classifications relate to each other. It would be of great interest to determine whether low-bursting and high-bursting pyramidal cell sub-populations uncovered by our ISICA method would relate to those distinct pyramidal cell types along the superficial-deep axis of the CA1/subiculum cell layer^30^,^47^ or along the dorsal-ventral axis (proximal-to-distal axis) distributed late-bursting and early-bursting pyramidal cells as observed from *in vitro* hippocampal slices^28^. It should be noted that burst may change spike form^49^,^50^. It seems that with well-isolated units, such changes may not significantly affect cell classifications, but will require further investigations.

Second, our ISICA method uncovered two distinct DA neuron subtypes from the mouse VTA region. Again, the classification membership remained invariant across different brain activity states (awake vs. sleep). More importantly, the optogenetic experiments have verified their DA cell identity. Surprisingly, the optogenetic experiments discovered that the irregular-spiking DA neurons had the latency at 5.4 ms upon light stimulation. On the other hand, the regular-spiking DA neurons had a shorter latency (3.7 ms). It raises an important question as to their underlying cellular, molecular and circuitry mechanisms. Future experiments will be needed to address these questions.

Third, we further investigated the relationship between the ISICA-based cell classification and functional significances of those cell subtypes. By examining how CA1 pyramidal cells would respond to ketamine-induced anesthesia or how DA neurons encode fearful information, we demonstrated that ISICA-classified cell subtypes indeed exhibit distinct encoding properties. In the case of the CA1 pyramidal cells, Ketamine, an uncompetitive NMDA receptor antagonist, selectively increased Delta power of the low-bursting pyramidal cell subtype. This may well reflect the distinct NMDA receptor subunit compositions on these cells. It is also possible such differential responses may result from a non-NMDA receptor mechanism, because ketamine also acts on the nicotinic and muscarinc Ach receptors, or opioid receptors^33^.

In the case of DA neurons, we uncovered that the ISICA-classified DA neuron subtypes exhibited remarkably different responses to fearful stimuli. The reward-coding theories previously described that DA neurons would be inhibited by or would not respond to aversive stimuli^51^. In a good agreement with the reward-coding theory, our analysis reveals that the regular spiking DA neurons showed a temporal suppression in firing during the aversive events. In contrast, the irregular-spiking DA neurons increased firing during earthquake or elevator-drop. Yet this observation is in consonance with the recent observations that putative DA neurons can respond to aversive stimuli, such as air-puff or electrical shock^14^,^24^,^40^,^52^. Our present study extended these observations by providing the first optogenetic evidence for confirming this type of DA neuron identity.

While DA neurons are known to fire action potentials phasically or tonically^53^,^54^, it is important to note that ISICA-classified DA cells both possess such properties upon fearful stimuli. It has been shown that phasic activities of VTA DA neurons depend on NMDA receptors^42^ and inputs from the nucleus pedunculopontinus^55^ and the laterodorsal tegmental nucleus^56^, whereas the tonic firing patterns may affect the postsynaptic mechanisms. Interestingly, based on temporal response dynamics during “*Early*” and “*Middle*” fear response periods, we noted that these two VTA DA neuron subtypes could be further divided into four subcategories (Figure S5a): 1) Subcategory-1a, Exc-Inh neurons (significant excitation during “*Early*” period and significant inhibition during “*Middle*” time-window period, blue stars in Figure S5b,c), biphasic, 2) Subcategory-1b, Exc-None neurons (significant excitation during “*Early*” period and no significant change during “*Middle*” period, blue circles in Figure S5b,c), monophasic, 3) Subcategory-2a, Inh-None neurons (significant inhibition during “*Early*” period and no significant change during “*Middle*” period, yellow circles in Figure S5b,c), monophasic, 4) Subcategory-2b, Inh-Exc neurons (significant inhibition during “*Early*” period and significant excitation during “*Middle*” period, yellow stars in Figure S5b,c) biphasic. Taken together, these results suggested the richness of VTA DA neuron subtypes.

Although the ISICA method is currently focused on pyramidal cells and DA neurons, the ISICA method holds the promise to profile different cell types in various brain regions. For instance, one can explore additional features that would profile vastly more complex forms of interneuron types^26^,^27^ and GABAergic neurons^13^. We envision that this ISICA method can be further combined with network oscillatory properties for providing finer classification (i.e. gamma, theta and ripple) among different brain or behavioral states^23^,^27^,^57^,^58^.

In summary, we described an unbiased computational classification method for profiling cell subtypes from *in vivo* neural spike datasets. This ISICA method should be highly complementary to other classification methods (i.e. cre/lox-mediated cell type-specific tracing, optogenetic manipulation and juxtacellular recording and labeling) for brain activity mapping. It can be particularly valuable to generate a census of neuron subtypes in the brain, especially under the context of cognition and behaviors.

## Methods

### Ethics Statement

All animal work described in the study were carried out in accordance with the guidelines laid down by the National Institutes of Health in the United States, regarding the care and use of animals for experimental procedures, and was approved by the Institutional Animal Care and Use Committee of Georgia Regents University (Approval AUP number: BR07-11-001) and Banna Biomedical Research Institute Animal Care and Use Protocol BRI-2458.

### Surgeries and *in vivo* recording in the hippocampal CA1 region

The CA1 units were recorded using the 96-channel tetrode recording array, as previously described^5^,^23^. The electrode bundles were positioned above the bilateral dorsal hippocampi (2.0 mm lateral to the bregma and 2.3 posterior to the bregma on the both right and left sides). After surgery, the mouse was allowed to recover for three to five days. Then the electrode bundles were advanced slowly toward the hippocampal CA1 region, in daily increments of about 0.07 mm until the tips of the electrodes had reached the CA1, as deduced from an assessment of high-frequency ripples, field potential, and neuronal activity patterns. At the end of the experiments, the mice were anesthetized and a small amount of current was applied to the recording electrodes in order to mark the positions of the stereotrode bundles. The actual electrode positions were confirmed by histological Nissl staining using 1% cresyl echt violet. The stability of the *in vivo* recordings was judged by waveforms at the beginning, during, and after the experiments.

Recordings were first carried out while the animals were in their home cages. Quiet awake episodes were manually assigned when the mice were awake and immobile. To identify slow-wave sleep, local field potentials were first band-pass filtered in theta band (4–12 Hz) and delta band (1–4 Hz), and then the ratio of the power in theta band to delta band was calculated. Two criteria were applied to extract the slow-wave sleep state: (1) Duration of an epoch was longer than 5 s, and (2) The ratio of the power during an epoch was greater than mean 5SD. For each mouse, the awake and SWS states were recorded for at least 15 minutes.

To produce ketamine-induced anesthesia, the animals were then injected with a 60 mg/kg ketamine and 0.5 mg/kg Domitor cocktail mixture via *i.p.*, and the animals lost the righting reflex in a few minutes. Neural spike activities were recorded for at least 50 minutes under anesthetized state. Neural spike data recorded from the fully anesthetized state starting from 10 to 45 minutes after the ketamine injections were selected for the present analysis.

### Surgeries and *in vivo* recording in the VTA

A 32-channel (a bundle of 8 tetrodes) electrode array was constructed, as previously described^24^. Two aversive stimuli, elevator-drop free fall (from 30 cm) and earthquake-like shake (for 0.5 sec), were used with an interval of typically one to two hours between sessions. We used either a square (10 × 10 × 15 cm) or round chamber (11 cm in diameter, 15 cm in height) for the free fall events. We used a round chamber (12.5 cm in diameter, 15 cm in height) for the shake events as described previously^24^. Dopamine neurons listed in the present study were stably recorded and well-isolated during both free fall and shake events, without temporary loss of unit or noise/artifact contamination.

### Data processing and spike sorting

The neuronal activity was recorded by a Plexon multi-channel acquisition processor system. The recorded spike activities were collected as described previously^23^. The well-separated neurons were assessed by “Isolation Distance” and “*L*_*ratio*_”^59^. In the present analysis, neurons whose “Isolation Distance” >15 and “*L*_*ratio*_” <0.7 were selected for further analysis. Isolated units were classified as either putative excitatory pyramidal cells or interneurons based on their characteristic firing activity including waveforms, inter-spike intervals, and firing rates^60^. In general, pyramidal cells fire at lower rates (<5 Hz) and have broader waveforms (>300 *μ*s); whereas, interneurons show the higher rates (>10 Hz) and have relatively narrower waveforms (<250 *μ*s) (Figure S6). Additionally, Pyramidal cells occasionally fire complex-spike bursts of two to seven spikes at 3–10 ms inter-spike intervals, reflected by their characteristic autocorrelograms and inter-spike interval histograms. A total of 70 well-separated hippocampal CA1 putative pyramidal cells (22, 23 and 25 from each mouse) were identified based on these criteria for the ISICA analysis. The waveform widths and mean firing rates of these 70 putative pyramidal cells were 338.433 ± 4.685 *μ*s and 2.717 ± 0.242 Hz, respectively.

### The Gamma distribution model of inter-spike interval histogram

Gamma distribution model was applied to describe the overall shape of neuron’s ISIH^16^. Gamma distributions were defined by a shape parameter *k* > 0 and a scale parameter *θ* > 0, and Gamma probability density functions for spike trains were defined by
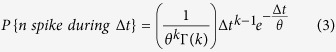
where Γ(*k*) was the Gamma function.

We fitted gamma distribution for ISIH of each neuron. Shape parameter *k* and scale parameter *θ* were estimated using the maximum likelihood estimates (MLEs) method, which selected values of the parameters that produced a gamma distribution for the histogram of spike-interval with minimal errors. To avoid the under-sampled error due to few data points, only ISIHs with >250 ISI were fitted in the present analysis.

### Unbiased clustering analysis based on the *k*-means clustering and the “jump method”

Unbiased clustering analysis was conducted by combining the *k*-means clustering method and the jump method^22^ to unbiasedly uncover neuron subtypes. In the present analysis, the distance measurement used by *k*-means clustering was the cosine distance, which was defined as one minus the cosine of the included angle between vectors. Each centroid was the mean of the points in that cluster. The “jump method” was introduced to determine the optimal number of clusters within the data. Since the *k*-means partitioning may depend upon the starting points used, the *k*-means algorithm is repeated a number of times (10,000 times) with different starting conditions, and a mean distortion for each prescribed value of *k* is obtained. A distortion curve is then generated by plotting the mean distortion as a function of *k*. The distortion tends to decrease as the number of clusters is increased, and this is transformed into an increase by raising the distortion to a negative power, *Y*, as shown in Fig. 1. Because the distortion drops when the correct number of clusters is used and remains roughly constant when even more clusters are employed, the transformed distortion exhibits a sudden increase, or jump, at the correct value of *k*. If one examines the size of the jumps in the transformed distortion, the largest jump is thereby an indication of the proper number of clusters. Thus, using spike data from the awake and slow-wave sleep states, the optimal number of clusters was unbiasedly chosen by the jump method.

We used cluster index to measure the quality of clustering results. Cluster index^28^ is defined as the ratio of the sum of the square distances from each point to its cluster center and the sum of the square distances from each point to the overall mean. A million cluster index values were calculated by repeatedly drawing samples from a single multivariate Gaussian distribution, and the *p* value was defined as the likelihood that the simulated data had a cluster index greater than the experimental data. The simulated results showed that the spike data were best represented by two clusters (*p* < 1E-10 for all analysis).

### Local field potential spectral analysis

The local field potential power density was calculated in a range of 0.025–25.0 Hz with 0.025 Hz intervals for the recording dataset of hippocampal CA1 region. The fast Fourier transform was applied to the EEG signal, the resulting frequency resolution was 0.025 Hz, and the frequency bins less than 1 Hz were discarded due to the sensitivity of these bins to noise. Then frequency bins were averaged for 0.5 Hz bins, and each value was expressed as a relative percentage of the total.

### Optrode and *in vivo* photo-stimulation

A modified 64-channel (two bundles of 8 tetrodes for bilateral recording), movable optrode array was constructed as follows: Each tetrode was made by twisting four 10-*μ*m diameter Platinum wires (90% Platinum 10% Iridium, California Fine Wire). Cladding was removed from two 200-*μ*m core, 037NA standard hard cladding multimode fiber (ThorLabs), and the optical fibers were placed 1-mm apart in a Microdrive base. Eight tetrodes were threaded into separate polymide tubes and placed adjacent to each optical fiber to create a bilateral bundle, with the tip of the optical fibers 300 to 600-*μ*m above the recording tetrodes. The bundle was then secured to a moveable screw nut on the Microdrive base.

Experiments were conducted using adult male (25–35 g) DAT-Cre mice (eDAT2) backcrossed to C57BL/6J mice. Adeno-associated virus (AAV, serotype 9) coding for Cre-inducible channelrhodopsin-2 (ChR2) and green fluorescent protein (GFP) under the control of actin promoter were injected bilaterally into each VTA (3.4-mm posterior to bregma, 0.5-mm lateral, and 4.7 to 5-mm ventral to the brain surface). Mice were allowed to recover for nine to 12 days before starting any experiment to allow for ChR2 expression in VTA dopamine neurons.

Prior to stimulation, PM100D (ThorLabs) was used to measure the light intensity. Optical fibers were connected to a blue laser (473 nm, diode-pumped solid state, Shanghai Dream Lasers Technology Co.). Trains of 20 Hz and 10 Hz stimulations (5 ms per pulse, 20 pulses per train) were delivered in separate trials using a PulsemasterA300 before and after conducting experiments each day.

### Test of statistical significance

In the present study, one-way ANOVA analysis and Tukey *post hoc* tests were conducted for the comparisons of multiple means. Student’s *t*-test was used here to assess whether two sets of data were significantly different from each other. In all figures that contained statistical significant test results, one asterisk denoted that the *p*-value is in the range of 0.05-0.01, two asterisks denoted that the *p*-value is in the range of 0.01-0.001, three asterisks denoted the *p*-value is less than 0.001. Data were represented as mean ± SEM.

## Additional Information

**How to cite this article**: Li, M. *et al.* Computational Classification Approach to Profile Neuron Subtypes from Brain Activity Mapping Data. *Sci. Rep.* **5**, 12474; doi: 10.1038/srep12474 (2015).

## Supplementary Material

Supplementary Information
