# Supplementary material for: Computational Classification Approach to Profile Neuron Subtypes from Brain Activity Mapping Data
Source: Sci Rep. 2015 Jul 27;5:12474. doi: 10.1038/srep12474 (PMC4515637; doi:10.1038/srep12474)
Supplement: Supplementary Information [file srep12474-s1.pdf]

Computational Classification Approach to Profile Neuron Subtypes from Brain Activity Mapping Data

Meng Li,[1,+] Fang Zhao,[1,+] Jason Lee,[1] Dong Wang,[1] Hui Kuang,[1,2] and Joe Z. Tsien[1,2,*]

[1]Brain and Behavior Discovery Institute and Department of Neurology, Medical College of Georgia, Georgia Regents University, Augusta, GA 30912, USA.

[2]The Brain Decoding Center, Banna Biomedical Research Institute, Xi-Shuang-Ban-Na Prefecture, Yunnan Province 666100, China.

[+]Co-first author

[*]Correspondence should be addressed to Joe Z. Tsien, Brain and Behavior Discovery Institute and Department of Neurology, Medical College of Georgia, Georgia Regents University, Augusta, GA 30912, USA. E-mail: jtsien@gru.edu.

Figure S1

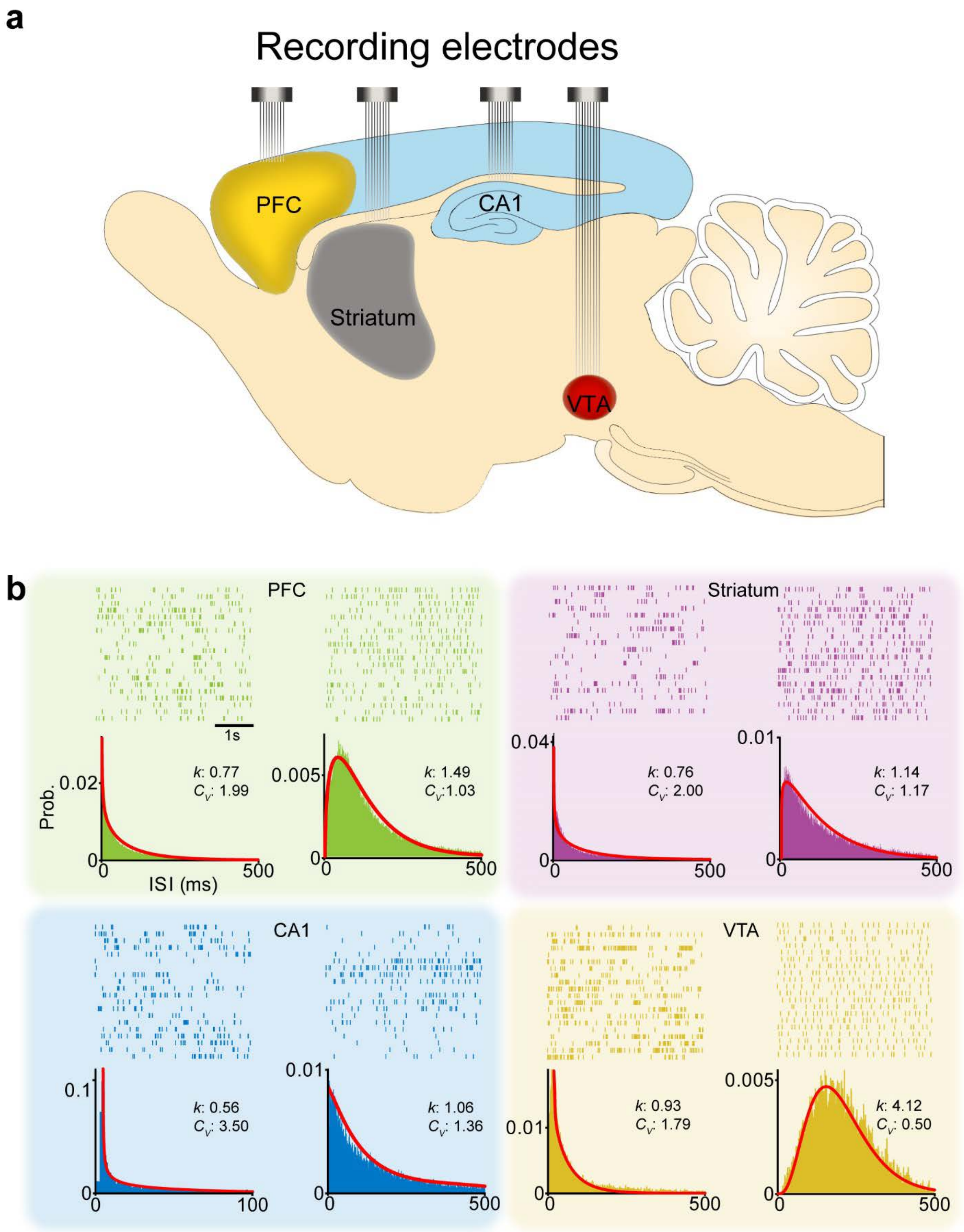

(a) Neural activity patterns of the primary neuron types were recorded in four well-studied brain regions, that is, pyramidal cells in the ACC, medium spiny neurons in the striatum, pyramidal cells in the CA1 region of hippocampus and DA neurons in the VTA. **(b)** Neural activity patterns of these four primary neuron types show massive variations, and these variations can be well described by k and c_v. Red curves are the Probability Distribution Function of Gamma distribution for each neuron.

Figure S2

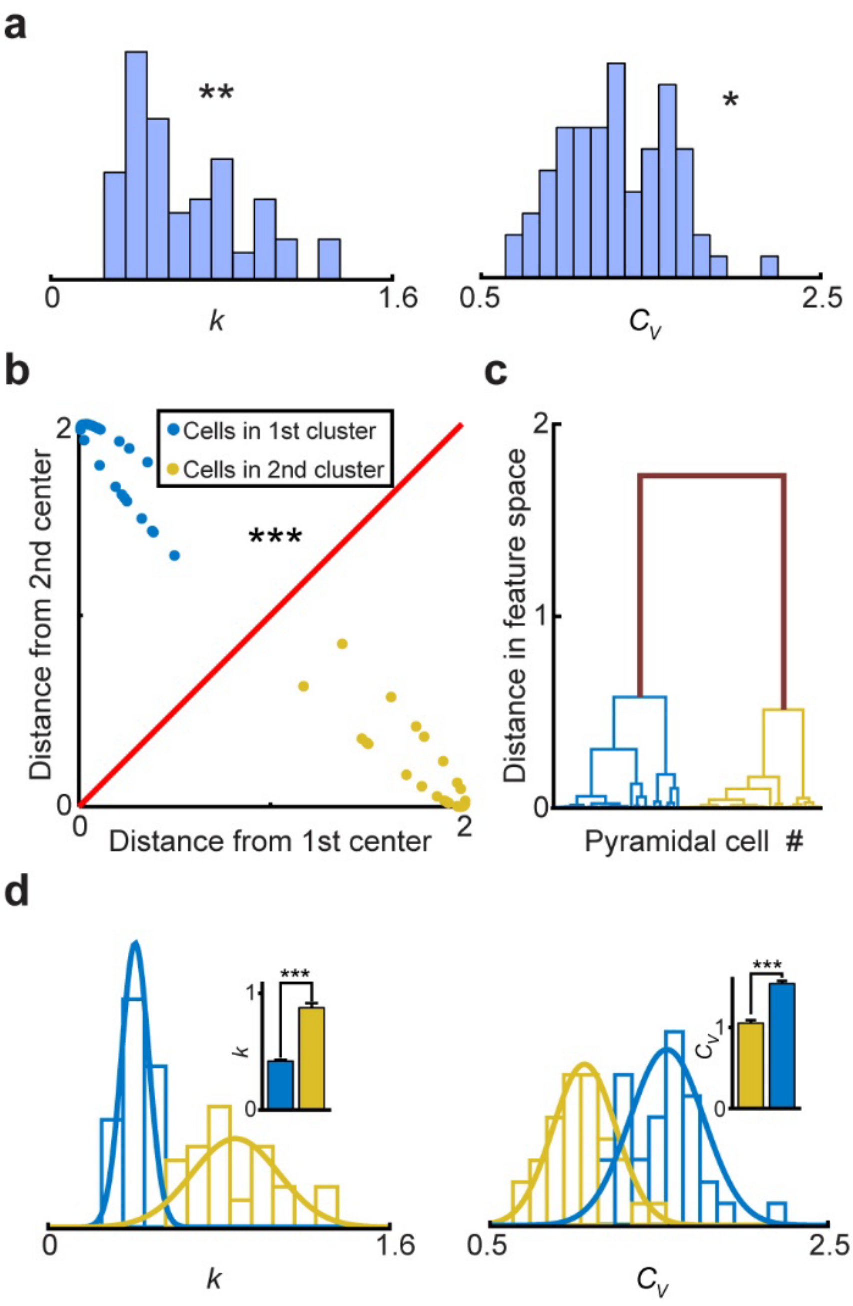

Profiling the hippocampal CA1 pyramidal cells based on neural activity patterns under the SWS state. (a) Distributions of *k* and c_v under the SWS state. *p* values from the D'Agostino and Pearson omnibus normality test indicated that there are discrete sub-populations within hippocampal CA1 pyramidal cell population. **(b)** Distances from two cluster centers revealed a significant separation of two pyramidal cell subtypes. **(c)** A hierarchical clustering analysis showed that the inter-cluster distance of two clusters was significantly higher than the intra-cluster distance. **(d)** Distributions of *k* and c_v for two pyramidal cell subtypes. The bar graphs showed that these two pyramidal cell subtypes had significant differences in *k* and c_v.

Figure S3

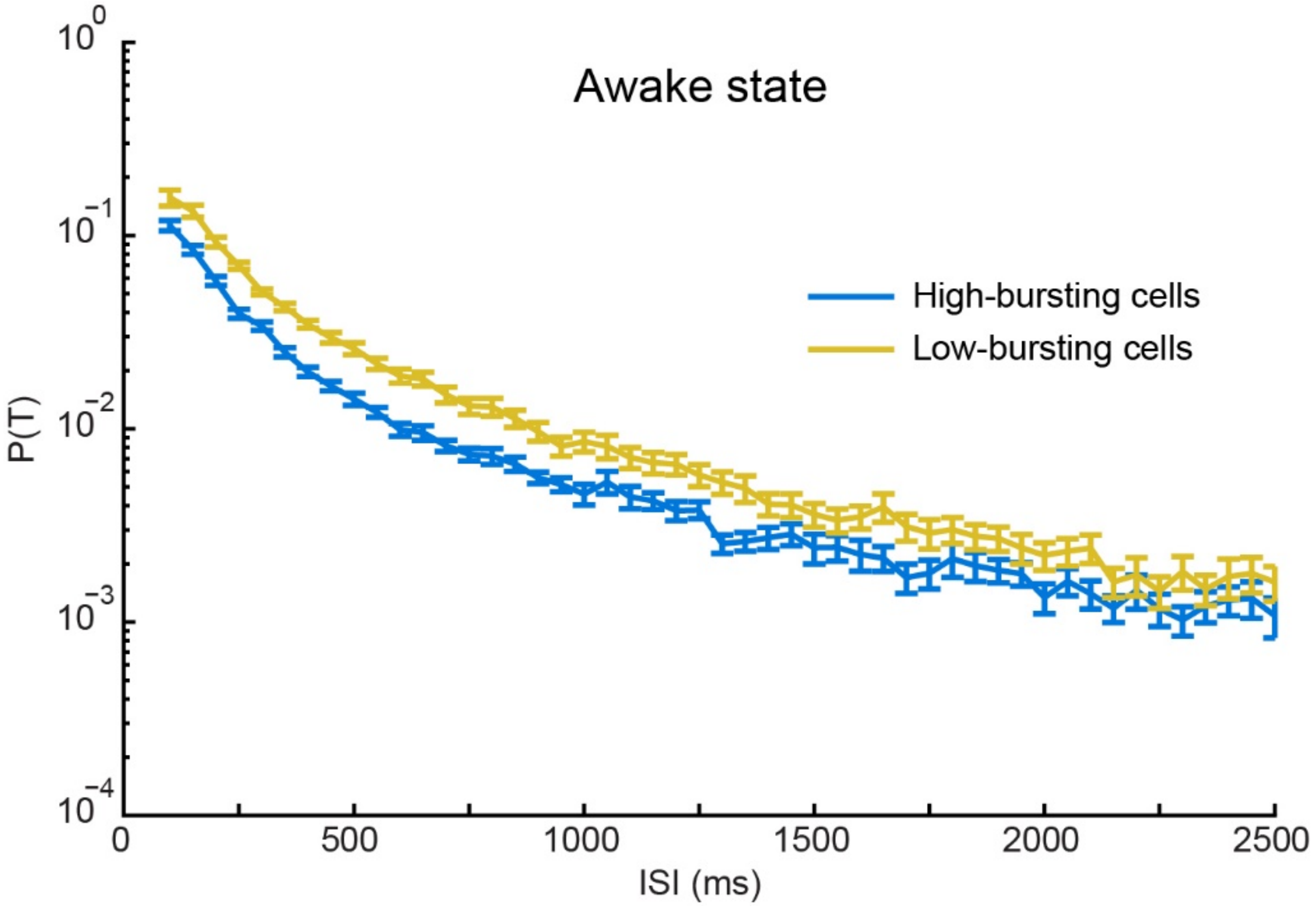

The ISIHs of both pyramidal cell sub-populations showed no significant difference under the quiet awake state.

Figure S4

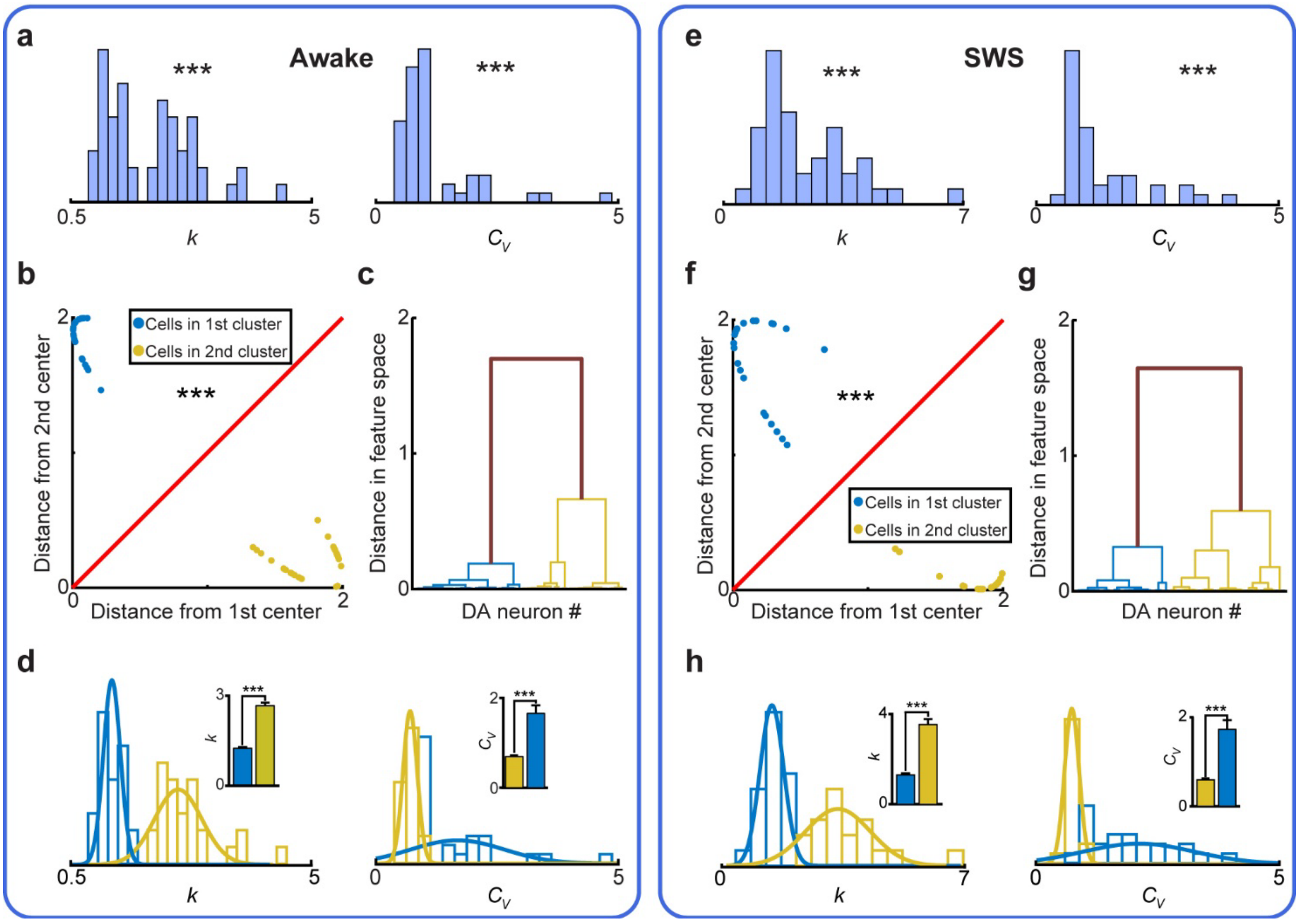

Profiling the VTA DA neurons based on neural activity patterns under the awake and SWS state. (a) Distributions of *k* and c_v under the awake state. *p* values from the D'Agostino and Pearson omnibus normality test indicated that there are discrete sub-populations within VTA DA neuron population. **(b)** Distances from two cluster centers revealed a significant separation of two DA neuron subtypes. **(c)** A hierarchical clustering analysis showed that the inter-cluster distance of two clusters was significantly higher than the intra-cluster distance. **(d)** Distributions of *k* and c_v for two DA neuron subtypes. The bar graphs showed that these two DA neuron subtypes had significant differences in *k* and c_v. **(e)** Distributions of *k* and c_v under the SWS state. **(f)** Distances from two cluster centers revealed a significant separation of two DA neuron subtypes. **(g)** The inter-cluster distance of two clusters was significantly higher than the intra-cluster distance. **(h)** Distributions of *k* and c_v for two DA neuron subtypes. The bar graphs showed that these two DA neuron subtypes had significant differences in *k* and c_v.

Figure S5

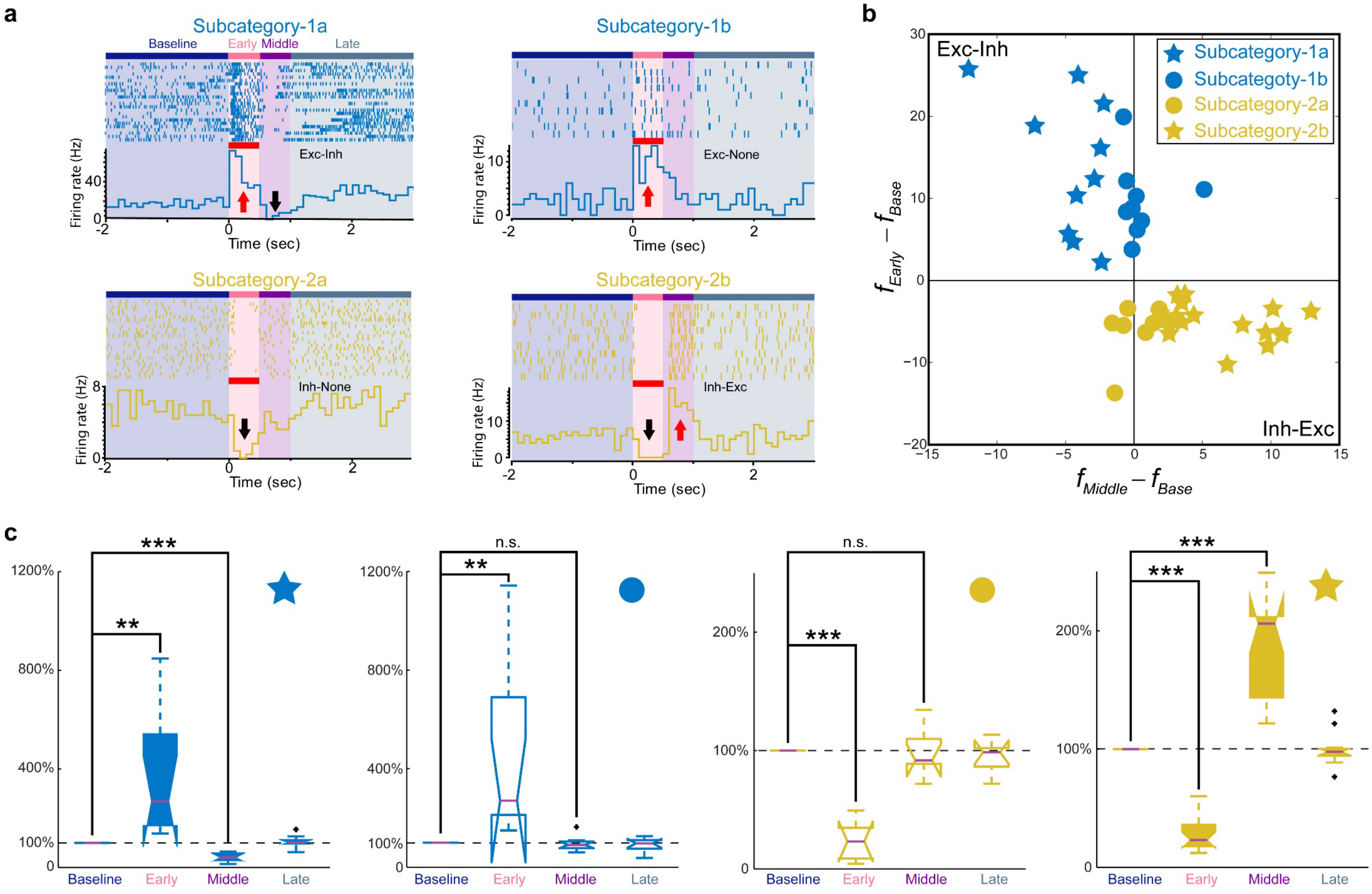
a
Subcategory-1a
Baseline
Early
Middle
Late
Exc-Inh
Firing rate (Hz)
Time (sec)
Subcategory-1b
Exc-None
Subcategory-2a
Inh-None
Subcategory-2b
Inh-Exc
b
Exc-Inh
Inh-Exc
Subcategory-1a
Subcategoty-1b
Subcategory-2a
Subcategory-2b
$f_{Early} - f_{Base}$
$f_{Middle} - f_{Base}$
c
***
**
n.s.
1200%
800%
400%
200%
100%
0
Baseline
Early
Middle
Late

Diverse responses of VTA DA neurons upon aversive stimuli. (a), The responses of four representative VTA DA neurons upon aversive stimuli, shown as the format of peri-event raster. The color bars above denote four different periods, the red/black arrows indicate that the firing rates of neurons increase/decrease significantly. Red bars in the middle of peri-event raster plots show the time periods of stimuli (0.5s earthquake). **(b)**, Scatter plot of responses of four DA neuron subcategories upon aversive stimuli. **(c)**, One-way ANOVA analysis of the mean firing rates of four VTA DA neuron subcategories during four periods. The firing rates of DA neurons during "*Early*," "*Middle*" and "*Late*" periods were normalized as the percentages of the firing rates during "*Baseline*" period.

Figure S6

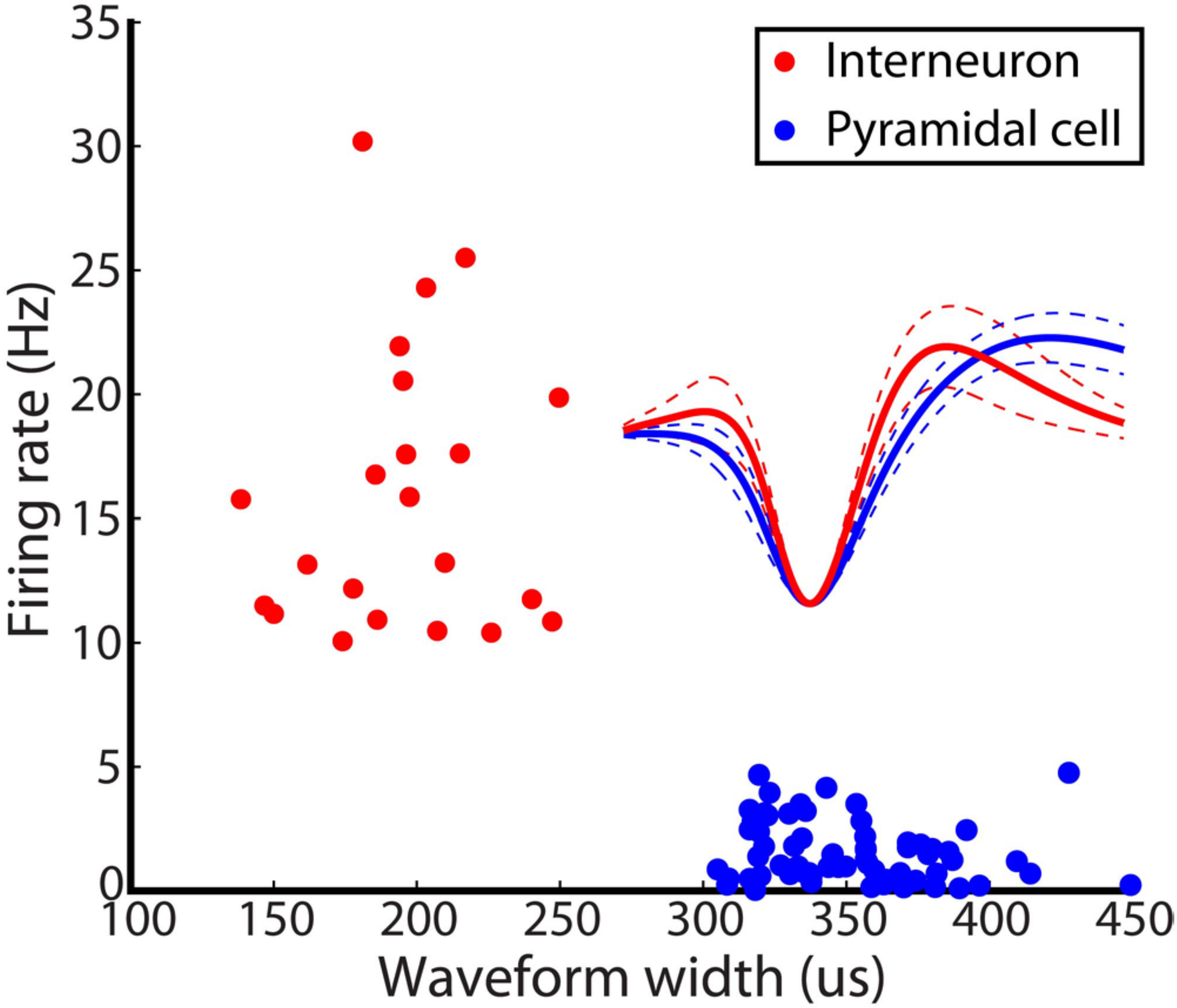

Classification of CA1 pyramidal cells and interneurons based on their firing rates and waveform width. The top-right subplot shows the waveform of pyramidal cells and interneurons, dashed lines denote SD.

Figure S7

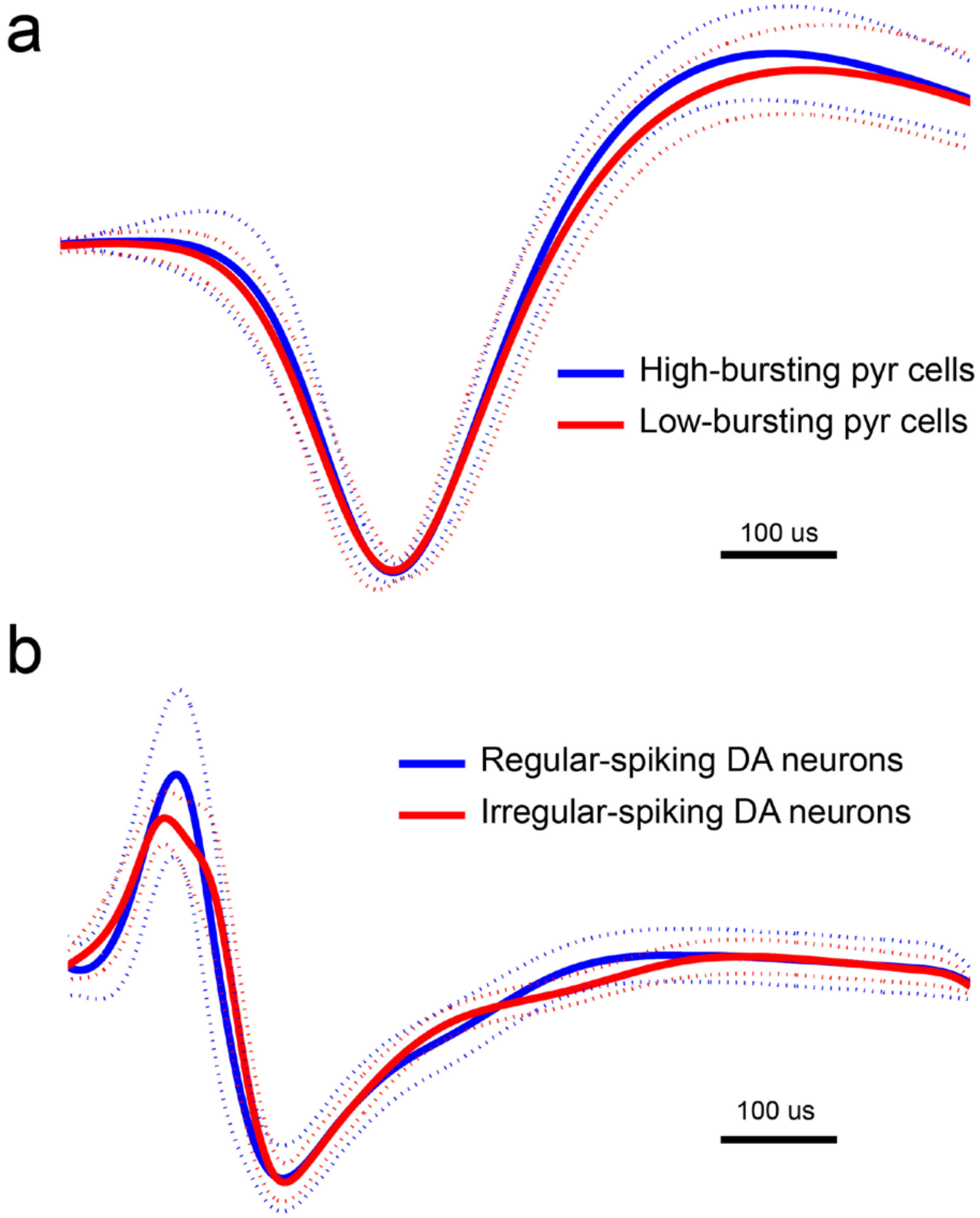

Waveforms of neuron subtypes. (a), Mean waveforms of two CA1 pyramidal cell subtypes. **(b)**, Mean waveforms of two VTA DA neuron subtypes. Dashed lines denote SD.
